# Alzheimer's disease: An evolving understanding of noradrenergic involvement and the promising future of electroceutical therapies

**DOI:** 10.1002/ctm2.397

**Published:** 2021-05-01

**Authors:** Cody Slater, Qi Wang

**Affiliations:** ^1^ Department of Biomedical Engineering Columbia University New York New York USA; ^2^ Vagelos College of Physicians and Surgeons Columbia University New York New York USA

**Keywords:** Alzheimer's disease, electroceuticals, neural stimulation, the locus coeruleus – norepinephrine (LC‐NE) system

## Abstract

Alzheimer's disease (AD) poses a significant global health concern over the next several decades. Multiple hypotheses have been put forth that attempt to explain the underlying pathophysiology of AD. Many of these are briefly reviewed here, but to‐date no disease‐altering therapy has been achieved. Despite this, recent work expanding on the role of noradrenergic system dysfunction in both the pathogenesis and symptomatic exacerbation of AD has shown promise. The role norepinephrine (NE) plays in AD remains complicated but pre‐tangle tau has consistently been shown to arise in the locus coeruleus (LC) of patients with AD decades before symptom onset. The current research reviewed here indicates NE can facilitate neuroprotective and memory‐enhancing effects through β adrenergic receptors, while α_2A_ adrenergic receptors may exacerbate amyloid toxicity through a contribution to tau hyperphosphorylation. AD appears to involve a disruption in the balance between these two receptors and their various subtypes. There is also a poorly characterized interplay between the noradrenergic and cholinergic systems. LC deterioration leads to maladaptation in the remaining LC‐NE system and subsequently inhibits cholinergic neuron function, eventually leading to the classic cholinergic disruption seen in AD. Understanding AD as a dysfunctional noradrenergic system, provides new avenues for the use of advanced neural stimulation techniques to both study and therapeutically target the earliest stages of neuropathology. Direct LC stimulation and non‐invasive vagus nerve stimulation (VNS) have both demonstrated potential use as AD therapeutics. Significant work remains, though, to better understand the role of the noradrenergic system in AD and how electroceuticals can provide disease‐altering treatments.

## Graphical abstract

In this review, we provide a brief overview of several of the most thoroughly researched pathogenic hypotheses for Alzheimer's disease (AD) and assess their clinical impact to‐date. We focus specifically on recent research into the role of the locus coeruleus norepinephrine (LC‐NE) system, in both AD pathogenesis and symptom exacerbation, as well as the potential to use advanced neural stimulation techniques as a novel therapeutic option in the earliest stages of neuropathology.

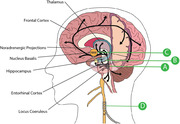

## 1 INTRODUCTION

The clinical syndrome of dementia is characterized by the slowly progressive decline of two or more cognitive domains, including but not limited to language, memory, executive function, personality, or behavior.^1^ Alzheimer's disease (AD) plays a significant role in the clinical presentation of dementia, accounting for up to 80% of all dementia diagnoses.^2^ In the United States alone, it is estimated that up to 4.7 million individuals over the age of 65 suffered from AD in 2010, and it is projected that 13.8 million Americans will be living with AD by 2050.^3^ AD was also ranked as the sixth leading cause of death in the United States in 2010, with more than 84,000 deaths attributed to the disease, a 3.7% increase from the year prior.^4^ The overall economic burden of AD is also significant, leading to an annual cost of $100 billion, third in the United States behind heart disease and cancer.^5^ The impact is even more severe globally, with up to 24 million cases in 2012. That number is predicted to double every 20 years, until at least 2040.^6^

In AD, dementia is currently viewed as the end stage of decades of neuropathological changes, which lead to a clinical manifestation that can range from asymptomatic, to a mild cognitive impairment (MCI), to a full amnestic syndrome with resultant loss of function.^7^, ^8^ The presentation and progression of this spectrum is highly variable between individuals, with a significantly higher prevalence in women, that has not been fully explained (for a review, see Nebel et al).^8^, ^9^, ^10^, ^11^, ^12^, ^13^, ^14^, ^15^ There is currently no effective method to prevent AD, and therapy is almost exclusively targeted towards symptomatic treatment. A large volume of work exists characterizing the underlying pathophysiology of AD, but a definitive consensus on the most promising therapeutic avenue remains elusive.^1^

The purpose of this review article is to give a brief overview of some of the most thoroughly researched hypotheses and assess their clinical impact to‐date. The intent is not to provide a complete review of the progress in each, but to briefly highlight how they have contributed to an understanding of the disease. Most of the attention of this review will be focused on recent research into the role of the noradrenergic system in both AD pathogenesis and symptomatic exacerbation, as well as the potential to use advanced neural stimulation techniques to offer novel therapeutic options targeting the earliest stages of neuropathology.

## 2 UNDERSTANDING THE PATHOPHYSIOLOGICAL PROGRESSION OF ALZHEIMER'S DISEASE

The general brain abnormalities found in Alzheimer's disease (AD) were first described by Alois Alzheimer in 1906.^16^ The disease is characterized by three major histopathological findings: brain atrophy, extracellular deposits of dense amyloid plaques, and intracellular cytoskeletal abnormalities such as accumulated neurofibrillary tangles.^17^ The neuronal death in AD is diffuse and markedly more severe than that seen in the normal aging process. The deposits of amyloid, a fibrillar peptide arranged in sheets, are associated with evidence of nearby inflammation, such as swollen axons and dendrites, along with reactive processes of surrounding astrocytes and microglia. Another common finding is amyloid accumulation in the cerebral vasculature. In diseased neurons that are still alive, filamentous tangles are observed in cell bodies and proximal dendrites. These tangles contain helical and 15 nm straight filaments.^17^

**HIGHLIGHTS**

- Locus coeruleus (LC) is the first brain structure to exhibit AD‐like pathology.
- Severe LC degeneration is a ubiquitous feature of AD, and the LC degenerates much earlier than symptoms are clinically apparent.
- Norepinephrine produced by the LC has an anti‐inflammatory influence in the brain.
- Direct LC stimulation and non‐invasive vagus nerve stimulation have both demonstrated potential as AD therapeutics.

The plaques and tangles constituting AD have been identified as typically localizing to specific brain regions.^17^, ^18^ Areas of susceptibility in AD include the locus coeruleus (LC), the nucleus basalis of Meynert (NBM), and the neocortex. The entorhinal cortex and hippocampus are also significantly impacted and likely contribute to early problems with declarative memory.^19^ The lateral entorhinal cortex, specifically, has been found to be dysfunctional in preclinical AD and can act as a source for the spread of disease to the parietal cortex and other regions of the brain.^19^ The NBM is one of the most heavily researched neuroanatomic regions in AD and has been found to be particularly vulnerable to neurofibrillary degeneration.

**FIGURE 1:**
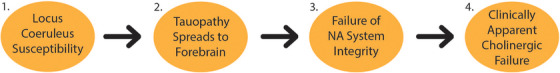
Pathway to noradrenergic dysregulation in Alzheimer's Disease: 1) Due to a combination of genetic and environmental factors, amyloid plaques and tauopathies occur in the Locus Coeruleus (LC) decades before symptom onset. 2) LC neuron count decreases and tauopathy spreads along anterior pathway to the forebrain and cortex during an asymptomatic period in middle‐age. Remaining noradrenergic (NA) neurons exhibit compensatory alterations. 3) NA system integrity is lost due to maladaptive LC changes. α and β AR expression alters across various brain regions, further disrupting connectivity. 4) Tauopathy spreads to the NBM. Hyperactive NA neurons may further inhibit the remaining cholinergic neurons. Widespread dysfunction occurs across multiple systems

The efforts to build a complete understanding of the temporal evolution of AD progression have remained limited by the reliance on post‐mortem autopsies to characterize the disease, though numerous efforts are ongoing to discover biomarkers that can be used to further characterize how AD spreads and to aid in early clinical diagnosis.^1^, ^8^, ^20^, ^21^, ^22^, ^23^, ^24^, ^25^ To‐date, a stereotyped progression has not been entirely described, though a general outline for how AD pathology can be expected to progress is slowly being revealed. The earliest preclinical findings in AD appear to be amyloid accumulation and tauopathy in the brainstem, specifically the locus coeruleus (LC; Figure 1).^26^, ^27^, ^28^, ^29^ This accumulation can occur up to several decades before clinical manifestations, suggesting a threshold, second trigger, or loss of functional reserve are necessary for disease progression. As the Braak stage, a systematic method for staging AD, increases, a linear decrease in LC volume, not associated with normal aging, has been described, beginning as early as the fourth decade of life in sporadic AD patients.^28^ From this early finding, tau pathology seems to spread to the dorsal raphe nucleus, entorhinal cortex, and perirhinal cortex, suggestive of anterior pathway susceptibility.^19^, ^30^ Layer II of the lateral entorhinal cortex appears to be the source of further progression to downstream synaptic circuitry, including the dentate gyrus and the cingulate cortex.^30^, ^31^

Eventually, accumulation of toxic tauopathies in cholinergic neurons of the NBM precipitates dramatic neuronal degeneration, followed by the progression of a more rapid decline in cognitive function (Figure 1).^19^, ^32^, ^33^, ^34^ Though the evidence now suggests that the NBM is primarily implicated in symptomatic disease, it is still debated whether this pathology is primary or secondary. Multiple autopsy studies have shown that most cholinergic neurons in this region remain unaffected by tauopathy in mild AD but are extensively involved in severe AD, suggesting that NBM degeneration is likely a late‐stage finding following years of subclinical changes in other regions of the brain.^35^, ^36^ Following NBM degeneration, there is then spread of this tauopathy to the neocortical areas to which the NBM projects.^33^, ^37^ This final stage is associated with the classic cognitive deficits and bulk brain atrophy of AD.

Though the extracellular accumulation of amyloid plaques and the intracellular presence of neurofibrillary tangles were the first features identified in AD, it is now also recognized that synaptic degeneration, hippocampal neuronal loss, and aneuploidy are important features that may provide more primary contributions than originally suspected.^38^ There is also still much debate about whether the amyloid plaque and neurofibrillary tangles themselves lead to the symptoms of dementia directly or are the result of a broader pathologic process. While plaque‐centered hypotheses were traditionally the most thoroughly researched, interest has turned towards the heavily phosphorylated tau protein that comprise the neurofibrillary tangles. A summary of this progression and the resulting clinical benefits is shown in Table S1 and expanded upon in the section below. To‐date, results remain inconclusive.^39^

## 3 LEADING HYPOTHESES

### 3.1 Cholinergic hypothesis

The cholinergic hypothesis is the oldest theory underlying AD pathogenesis and builds off the observation that choline acetyltransferase (ChAT) activity is greatly reduced at synapses in the amygdala, hippocampus, and cortex of AD brains,^40^, ^41^, ^42^, ^43^ resulting in a corresponding cholinergic failure and impairment of memory, attention, and learning.^44^, ^45^ The projections to the cerebral cortex of presynaptic cholinergic neurons in the nucleus basalis of Meynert (NBM) appear to specifically undergo profound degeneration in late AD.^43^, ^46^, ^47^ This leads to a subsequent loss of nicotinic receptors in the cerebral cortex and muscarinic M2 receptors, both of which are predominately pre‐synaptic.^48^, ^49^, ^50^ Decreases in M1 receptors in the dentate gyrus and pyramidal neurons in layers III and V of the parahippocampal cortex have also been demonstrated in AD patients.^51^, ^52^, ^53^ This body of research has led to the only clinically relevant treatment thus far for AD.

Cholinesterase inhibitors, pharmacological agents that reduce the degradation of synaptic acetylcholine, have been shown to have a consistent, though marginal, increase in functional outcomes over placebo.^48^, ^54^ It has been more than 20 years since the first cholinesterase, tacrine, was FDA approved, marking the last major milestone in AD therapeutics.^55^ The current FDA‐approved cholinesterase inhibitors include rivastigmine, donepezil, and galantamine. Though they appear to result in some histological changes in AD patients, clinical trials have shown efficacy only in minor symptom improvement, with no underlying impact on disease course or progression.^48^ Though disappointing in a clinical context, successful combination treatment in the future will likely include a cholinesterase inhibitor for patients that are already symptomatic.

Novel interventional approaches to halting or preventing cholinergic failure are in the early stages and are not currently being widely pursued. Researchers in Germany have performed small Phase I clinical trials using NBM bilateral, low frequency, deep brain stimulation (DBS) in six patients, four of which were considered responders on the basis of stable or improved primary outcome parameters 12 months after surgery. The study subsequently concluded NBM‐DBS is technically feasible and well‐tolerated.^56^ Additional follow‐up results support disease stabilization.^57^ Additional research on NBM‐DBS for other forms of dementia have shown results similar to those in AD patients in Germany.^56^, ^57^, ^58^, ^59^, ^60^, ^61^, ^62^, ^63^, ^64^, ^65^, ^66^, ^67^, ^68^, ^69^, ^70^, ^71^, ^72^, ^73^ A summary of the most relevant work is included in Table S2A. The mechanisms and overall impact of NBM‐DBS on disease pathophysiology are not well‐studied though,^63^, ^74^ and more investment is needed in this area to gain better control of the clinical response to NBM‐DBS.^75^ Efforts in this area may prove limited for long‐term applicability, depending on whether NBM degradation proves to be a primary or secondary characteristic of clinically apparent AD. Given the importance of acetylcholine release in memory formation, however, electroceutical manipulation of a dysfunctional cholinergic system offers a promising avenue for further exploration in the pursuit of any clinically relevant treatment.

### 3.2 Amyloid cascade hypothesis

The extracellular plaques seen in AD primarily consist of Aβ protein, which is created by processing the parent amyloid precursor protein (APP).^76^, ^77^, ^78^, ^79^ At picomolar levels, APP and Aβ are involved in normal neuronal functioning and synaptic plasticity modulation.^79^, ^80^, ^81^, ^82^ The position of the APP gene on chromosome 21 led to an interest in the connection of the presenile cognitive decline and amyloid plaque pathology seen in Trisomy 21,^45^, ^83^, ^84^, ^85^, ^86^, ^87^ however, subsequent work has demonstrated the limitations of applying the pathophysiology of this form of cognitive decline to the later‐onset, sporadic form of AD.^79^, ^88^, ^89^ This direction of research was important though because it led to the discovery of an autosomal dominant form of early‐onset AD, in which an APP gene mutation drives neurodegeneration.^90^ Though distinctly different from the more common sporadic AD, work in this area has demonstrated the neurotoxicity of Aβ and has contributed to the formulation of the amyloid cascade hypothesis.^90^, ^91^ This theory postulates that toxic plaques are the earliest pathology of AD.^91^, ^92^ The aberrant form of amyloid plaque induces the phosphorylation of tau protein through an imbalance in cellular signaling (Figure 3), the latter of which then spreads to local neurons via microtubule transport. The corresponding buildup of hyperphosphorylated tau protein results in cell death and gradual cognitive dysfunction.^77^, ^81^, ^91^, ^93^, ^94^, ^95^

Therapeutic targets to‐date include several monoclonal antibody therapies that target and remove Aβ from the brain, though none have resulted in a major improvement in cognitive function.^96^, ^97^ Alternative approaches to reducing Aβ are ongoing, with recent focus turning specifically to the most toxic Aβ oligomers.^1^, ^98^, ^99^, ^100^ The amyloid cascade hypothesis continues to undergo serial iterations, and the most recent adaptation hypothesizes that Aβ accumulation is part of a broader alteration in the homeostasis of APP‐related functions.^101^

### 3.3 Neurovascular hypothesis

The neurovascular hypothesis explores the vascular dysregulation that occurs in AD patients.^102^, ^103^ It has been known for nearly three decades that the cerebral microvasculature is damaged in AD, resulting in regional and laminar patterns of damage that follow neuronal degradation.^102^, ^104^, ^105^ Subsequent research has shown that Aβ protein can interact with vascular endothelial cells to produce excessive superoxide radicals.^102^, ^103^, ^106^, ^107^ These superoxide radicals then produce a litany of degenerative alterations and inhibit the production of nitric oxide, contributing to increased vasoconstriction and a reduction in the local blood supply, exacerbating further pathologies.^108^, ^109^, ^110^, ^111^, ^112^

The incomplete clearance of Aβ across the blood‐brain barrier (BBB) also contributes to eventual BBB degeneration and a chemical environment not conducive to cell survival.^107^, ^110^ Several studies have attempted to better understand the role of vascular function in AD, with some attention paid to the impact of hyperlipidemia and diabetes, which are also known to result in vascular endothelial damage, on AD prevalence and outcomes.^113^, ^114^ Results to‐date are not definitive, and no clinically relevant therapies addressing neurovascular dysfunction have been achieved, aside from a small risk reduction associated with preventative measures targeted at diabetes mellitus and artherosclerosis.^45^, ^115^, ^116^, ^117^

### 3.4 Mitochondrial cascade hypothesis

The idea behind the mitochondrial cascade hypothesis was first introduced in an attempt to explain a general pathophysiologic mechanism underlying the more common, sporadic form of AD.^118^ It postulates that an individual's basal rate of production of reactive oxygen species (ROS) is genetically determined and sets the pace at which acquired mitochondrial damage occurs.^118^ This eventually leads to three specifically defined events, termed a removal response, reset response, and replace response. Cellularly, this corresponds to an increase in Aβ generation due to the increase in ROS, compromised cells undergoing programmed cellular death, and neuronal progenitors unsuccessfully attempting to re‐enter the cell cycle leading to tau phosphorylation and aneuploidy.^118^, ^119^, ^120^, ^121^, ^122^, ^123^

There are several studies that seem to support this hypothesis, including findings that the middle‐aged children of AD mothers tend to utilize less glucose on fluorodeoxyglucose‐PET scans, exhibit more age‐associated brain atrophy, and perform worse on memory test performance than those of AD fathers.^124^ There is also evidence that functional mitochondria are required to mediate cellular damage when Aβ plaques are available. Though this hypothesis has not been definitively proven in humans, mitochondrial dysfunction appears to be a crucial feature in a multi‐factorial view of AD.^125^, ^126^, ^127^ Therapeutic trials targeting mitochondria have been mixed, with no AD‐approved drug yet appearing achievable, despite some mixed success with antioxidant treatments.^120^, ^121^, ^122^, ^124^, ^125^, ^128^, ^129^, ^130^, ^131^ Future work is likely to focus on more targeted delivery with nanoparticle therapy.^132^, ^133^, ^134^, ^135^

### 3.5 Tau propagation hypothesis

Highly phosphorylated tau (p‐tau) proteins comprise the majority of the neurofibrillary tangles (NFTs) seen in AD.^30^, ^93^, ^95^, ^136^, ^137^, ^138^ These proteins typically bind and help stabilize microtubules in axons.^139^, ^140^, ^141^ The function of these proteins is highly dependent on the phosphorylation state.^141^, ^142^ Hyperphosphorylation can expose a microtubule‐binding domain that enables self‐aggregation and oligomerization, eventually resulting in the formation of paired helical filaments, loss of axonal transport, and conversion to NFTs with further cellular disruption.^136^, ^137^, ^141^, ^142^, ^143^, ^144^, ^145^ It is currently thought that the tau oligomer intermediate is more important for disease progression than the resulting NFTs.^146^ Though the oligomerization process and formation of NFTs were originally viewed as a downstream effect of amyloid plaque accumulation, the levels of p‐tau correlate more closely with symptom severity and neuronal loss than Aβ plaque levels alone.^137^, ^147^, ^148^, ^149^ The pathological accumulation of p‐tau is now suspected to play a more primary role in disease progression with a direct, prion‐like spread and neuronal degeneration from the entorhinal cortex, to the perforant pathway, which links the cerebral cortex and hippocampus.^147^, ^150^, ^151^, ^152^ For these reasons, p‐tau targeted therapy has appealed as a promising avenue for the past decade.

Therapeutic investigation originally focused on microtubule stabilization, inhibition of the kinases responsible for phosphorylating tau, and direct inhibition of tau aggregation. These methods have mostly been abandoned due to their lack of efficacy or toxicity.^39^, ^153^ The approach to tau targeted treatment now centers on numerous immunotherapies that are still in the early stages of testing, but which have shown encouraging safety profiles thus far.^39^, ^153^, ^154^

## 4 THE ROLE OF THE NORADRENERGIC SYSTEM IN ALZHEIMER'S DISEASE

As evidenced by the brief review above, none of the proposed mechanisms has yet provided a full explanation for the pattern and distribution of AD pathology. As a result, many researchers have begun looking for alternative theories. Recent interest has turned to the role of the noradrenergic system, and the LC specifically, in AD pathology and symptomology. Studies have indicated an average reduction of 60% in LC cells compared to similarly aged controls, with LC degeneration occurring early in the time course of AD progression.^24^, ^26^, ^27^, ^155^, ^156^, ^157^, ^158^, ^159^, ^160^

### 4.1 Noradrenergic changes

**FIGURE 2:**
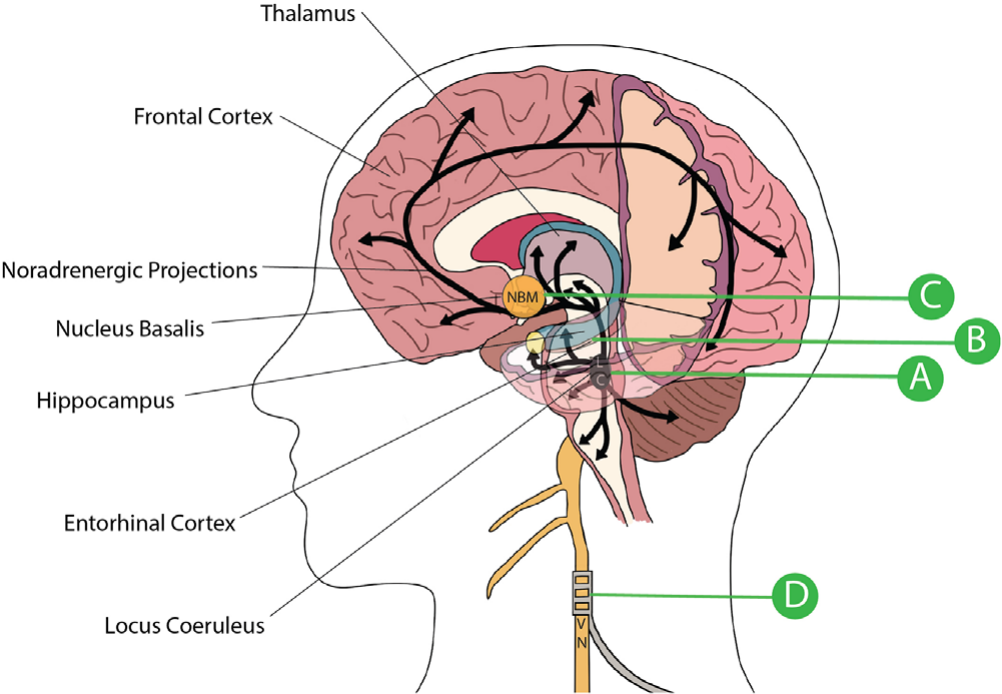
Role of electroceuticals in treating Alzheimer's disease. A, The Locus Coeruleus (LC) consistently exhibits the first pathology seen in AD and noradrenergic dysfunction exacerbates multiple aspects of disease progression. Chemogenetic manipulation has shown promising results. B, The entorhinal cortex is also significantly impacted in AD and likely contributes to early problems with declarative memory. Stimulation of the entorhinal cortex in animal models of AD have shown early promise in improving memory deficits. C, The Nucleus Basalis of Meynert (NBM) undergoes profound degeneration in late‐stage Alzheimer's disease (AD), leading to cholinergic dysfunction. Small clinical trials for NBM‐DBS have shown safety, tolerance, and modest levels of cognitive improvement or stabilization. D, Vagus Nerve Stimulation (VNS) is minimally invasive and has been proven to elevate NE and ACh levels in cortical and subcortical structures. Additionally, VNS alters microglial phenotypes and activates neuronal plasticity.

The noradrenergic system in the brain is critical for the regulation of many normal brain functions.^161^, ^162^, ^163^, ^164^, ^165^, ^166^, ^167^, ^168^ The primary source of norepinephrine (NE) to the forebrain originates in the LC, and selective deterioration of this nucleus has been shown to significantly disrupt multiple cognitive processes.^169^, ^170^ The LC projects to a wide area, including the hippocampus, cerebral cortex, basal forebrain, preoptic area, and hypothalamus (Figure 2), meaning any disruption in the function of the LC has widespread implications.^171^, ^172^ Norepinephrine is released from presynaptic terminals, with extracellular levels largely determined by catechol‐O‐methyltransferase (COMT) and monoamine oxidase (MAO) degradation, or reuptake into the presynaptic terminal by norepinephrine transporter (NET).^173^, ^174^, ^175^, ^176^, ^177^ The exact role of LC‐NE dysfunction in AD is not well understood, in part due to the numerous adrenergic receptor subtypes and their varying effects. A summary of what is known so far about how these receptors are altered in AD is presented in Table 1 (for a more thorough review, see Gannon et al).^170^, ^178^ Briefly, stimulation of the α_2A_ receptor has been correlated with amyloidogenesis, while diseased brains have been routinely found to exhibit decreased levels of the α_1A_ adrenergic receptor subtype in the prefrontal cortex.^179^, ^180^, ^181^, ^182^ Meanwhile, a decreased β/β_2_ receptor ratio has been observed in AD patients, with β_2_ blockade leading to a reduction in amyloidogenesis.^179^, ^183^, ^184^, ^185^, ^186^, ^187^, ^188^

**TABLE 1:** Currently understood role of noradrenergic receptor subtypes in Alzheimer's disease

Receptor	Subtype	Location	Role in Cognition	Changes in Alzheimer's Disease	
α_1_	α_1A_	Hippocampus	Improves spatial learning and memory^259^	↓ α_1A_ mRNA^180^	↓ α_1_ non‐subtype selective radioligands in the hippocampus^260^ and prefrontal cortex.^261^
	α_1B_	Amygdala	Improves fear learning^262^	–	
	α_1D_	Hippocampus	Improves working memory and attention^263^	↓ α_1D_ mRNA^180^	
α_2_	α_2A_	Hippocampus	Impairs spatial and fear learning^264^, ^265^, ^266^	↔ α_2A_ levels unchanged^267^	↑ α_2_ receptor density in remaining cortical membranes^268^ and dentate gyrus granule cell layer^178^, ^267^
		Prefrontal cortex	Improves working memory	↓ α_2A_ mRNA in layer II^180^	↑ α_2_ receptors in brain microvasculature innervated by LC^269^
		Cerebellar cortex	–	↑ α_2_ receptors in aggressive subgroup^270^	
	α_2C_	Hippocampus	–	↓ α_2C_ mRNA^267^	
β	β_1_	Hippocampus	Impairs spatial reference memory^178^	↑ β_1_ receptors^186^	↔ No consistent change in absolute level of β_1_ receptors in AD patients^271^
		Prefrontal cortex	–	↓ Decreased ratio of β/β_2_^186^	
		Putamen	–	↓ β_1_ receptors^186^	
	β_2_	Hippocampus	Impairs spatial reference memory^272^	↑ β_2_ receptors^186^	↓ β_2_ receptor density in cerebral microvessels^273^ ↓ Decreased ratio of β/β_2_^186^
		Prefrontal cortex	Improves memory retrieval^274^	↓ Decreased ratio of β/β_2_^186^	
		Thalamus	–	↓ β_2_ receptors^275^	
		Putamen	–	↔ No consistent change^186^	

Tauopathy in the LC occurs as early as adolescence, with neuronal degeneration progressing over a number of decades.^29^, ^31^, ^152^ In response to the overall loss of LC neurons, the surviving neurons display numerous compensatory changes, including sprouting additional axonal connections to the hippocampus and increasing the number of dendritic connections overall.^170^, ^180^ This keeps the number of connections relatively stable as AD pathology begins to progress but before symptoms are manifested clinically. It has also been shown that as AD develops, there is an associated increase in the enzyme tyrosine hydroxylase, which is responsible for the rate‐limiting step of norepinephrine synthesis, along with a decrease in the levels of norepinephrine transporter (NET).^180^, ^189^ As these disease processes continue there is mixed evidence on whether levels of norepinephrine (NE) are substantially altered in the brain. It is postulated that perhaps the regulatory mechanisms are able to compensate to keep extracellular levels of NE stable until late in the disease.^190^ This increased noradrenergic tone helps maintain extracellular NE input to the cortex during the early disease stages, while the overall tissue levels of NE in the hippocampus, temporal lobe, thalamus, and LC decline anywhere from 12% to 65%.^170^ At the time the most recent review on this topic was published, more consistent studies were needed to verify the extent of signaling dysfunction and adrenergic receptor involvement.^178^ See Table S2B for a more thorough summary of the relevant literature.

**FIGURE 3:**
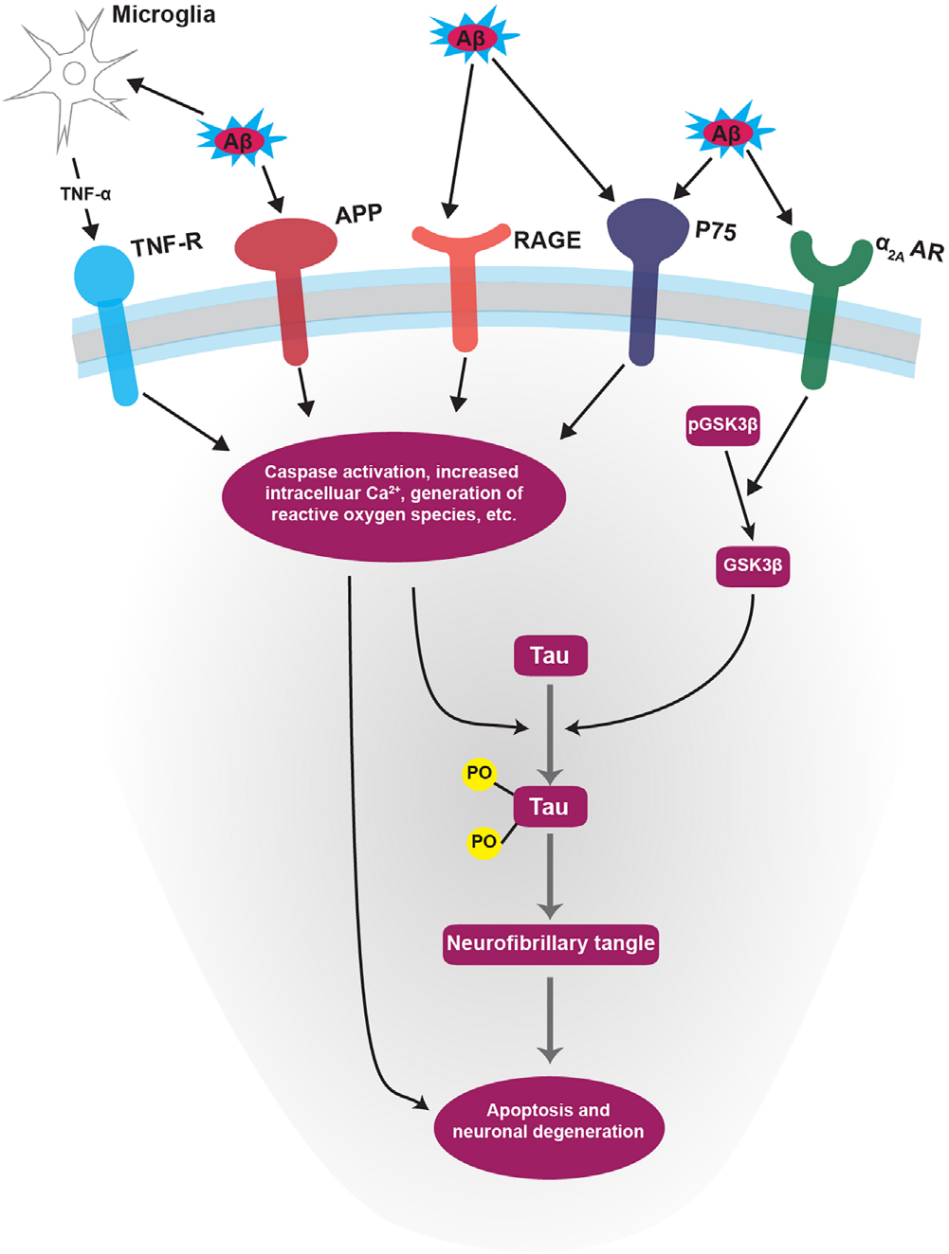
Alzheimer's disease signaling cascade. General signaling pathway underlying neuronal degeneration in Alzheimer's disease. Notably, adrenergic remodeling can influence pathologic activation of the α_2A_AR, which, coupled with the pro‐inflammatory loss of β‐AR integrity, may partially explain the accumulation of neurofibrillary tangles.

The most significant new contribution to understanding the involvement of various adrenergic receptor subtypes has focused on the α_2A_ adrenergic receptor (α_2A_AR), previously implicated in promoting amyloidogenesis (Figure 3).^181^ This work found that Aβ oligomers (Aβ_O_) act as an allosteric ligand of α_2A_AR. Aβ_O_ interaction aberrantly redirects NE‐induced α_2A_AR receptor signaling to activate the GSK3β/tau cascade, resulting in an increased intracellular signaling response and the subsequent hyperphosphorylation of tau (Figure 3).^190^ The α_2A_AR is present in many CNS structures, especially the LC, but is also present in other regions of the brainstem, midbrain, hypothalamus, and hippocampus.^182^ This supports the idea that AD‐specific neurodegeneration often begins with, and is a primary pathology of, the LC – before any other location. AD can then be thought of as affecting local neurons first and eventually spreading throughout the more classic regions of the brain. The disrupted cellular signal balance and dysregulation of microglial immune function create a complex interplay that eventually leads to the symptoms and pathology seen in AD.

Despite a limited understanding of the exact mechanisms underlying noradrenergic system dysregulation in the LC, several experiments have shown that a NE deficit exacerbates AD pathology, while NE supplementation appears to be beneficial. Ablation of noradrenergic neurons with DSP‐4 in AD transgenic mice increases Aβ deposition, alters adrenergic receptor subtype expression, and impairs spatial memory.^191^, ^192^, ^193^ Much of this effect appears to be related to the ability of the noradrenergic system to modulate the immune system, with selective ablation of the LC significantly impairing the ability for microglia to phagocytose Aβ.^192^ Additional experiments have shown that a DSP‐4 lesion of the LC also increases the levels of intracellular tau in the cortex of APP‐SL mice.^14^ In contrast, peripheral administration of a NE precursor in these animals restored some microglia function and increased Aβ clearance.^194^ NE appears to provide dose‐dependent protection to primary cortical and LC neurons from Aβ toxicity via the tropomyosin‐related kinase B (TrkB).^195^

The full scope of these findings point to a spectrum of illness in which the resulting imbalance of adrenergic receptors, in combination with increased demands on the remaining noradrenergic neurons and time‐dependent synaptic remodeling, lead to a positive feedback of amyloid accumulation, increased tau phosphorylation, immune system dysfunction, and neuronal death.^169^, ^170^, ^173^, ^178^, ^196^, ^197^, ^198^, ^199^, ^200^ More work should be pursued that can characterize the temporal and spatial characteristics of this disease process. Work that expands on the deleterious effects of a dysfunctional adrenergic system continues to be more compelling than that focusing on the late‐stage cholinergic changes mentioned previously. In fact, even when the disease has become clinically apparent, the most profound neuronal loss remains to be in the LC.^201^

Contributing to the complex involvement of this neurotransmitter system in AD, the LC is unique in that its neurotransmitter adds to the oxidative stress of its corresponding neurons.^158^, ^202^, ^203^, ^204^, ^205^ NE transporters reuptake the neurotransmitter after its release into the synaptic cleft which can result in cytoplasmic NE.^200^ This NE can autoxidize or be converted into a toxic metabolite by MAOs.^200^, ^206^ Additional metabolic stress occurs because of the near continuous activation of the LC, resulting in the reliance on mitochondrial oxidative phosphorylation.^205^ The human LC also synthesizes the granular pigment neuromelanin, which binds iron and other heavy metals from the blood; LC projections are exposed to an extensive microvascular surface area in the central nervous system.^207^ Norepinephrine itself also plays an important role in maintaining the BBB, meaning any dysfunction in NE can result in increased toxin exposure.^198^, ^208^, ^209^ In addition to the direct pathologies mentioned previously, dysfunction of LC neurons creates additional cerebral susceptibilities to further degradation. Therefore, an LC‐centric hypothesis would also build off the mitochondrial cascade hypothesis because the neurons in the LC are particularly prone to generating Aβ as a protective response.^200^

It is also worth noting that there is a growing interest in the role of gut‐brain axis dysfunction in AD.^210^, ^211^, ^212^ There has been some evidence to indicate that there is a link between the gut microbiota and the Aβ signaling pathway.^212^, ^213^, ^214^ The vagus nerve provides extensive innervation to the visceral organs and is a key bi‐directional mediator of inflammation in both the central nervous system and peripheral tissue.^210^, ^211^, ^214^, ^215^ The extent of the role of the enteric neurotransmitter system, and NE in particular, is poorly understood in relation to cognitive function, but is of significant interest due to the role stimulation of the vagus nerve plays in regulation of the peripheral immune system.^211^, ^215^, ^216^

### 4.2 Targeted noradrenergic therapies

The impact of noradrenergic neuron involvement in AD has generated much excitement at the prospect of utilizing NE‐targeted drugs to impede progression of the disease.^217^ Several animal models have shown that increasing NE holds the potential to treat both the neuropathological and cognitive decline of AD.^170^, ^190^, ^194^, ^195^, ^206^, ^217^, ^218^, ^219^, ^220^ In general, α_2A_ antagonism has been shown to reverse memory deficits in mice,^181^ and β receptor activation of the cAMP/protein kinase A signaling pathway reverses the toxic effects of Aβ_O_.^183^, ^184^, ^185^ There has also been promise in experiments demonstrating that attenuation of NET activity can markedly reduce beta amyloid deposition.^221^ Progress in building a successful theory behind the mechanisms responsible for improving memory performance remains complicated by the roles of the various adrenergic receptor antagonists (see Table 1).^187^, ^222^, ^223^ A possible explanation for this wide array of findings is that the compensatory changes seen in the LC as neurons began to die results in some signaling pathways and regions of the brain becoming overactivated, while others become suppressed. Broadly targeted NE treatments likely do little to correct this misbalance, especially late in the disease.^158^, ^202^, ^224^

Indeed, despite the growing body of evidence correlating disease severity most closely with noradrenergic neuron loss, drugs that act to increase synaptic NE have seen limited application in the clinical treatment in AD patients. MAO inhibitors, theoretically useful in increasing extracellular NE levels, have shown mixed results in clinical trials.^225^, ^226^ Selegiline, a MAO‐B inhibitor with proven neuroprotective benefits in Parkinson's, has exhibited very few significant treatment effects, though the studies have been small and a subsequent meta‐analysis has revealed a possible benefit on memory function.^226^ MAO inhibitors also come with a range of undesirable side‐effects and non‐selectively alter multiple other neurotransmitter systems.^227^, ^228^ This limits the maximum therapeutic efficacy, but, given the promise of norepinephrine in improving Aβ clearance and limiting cognitive decline, a tremendous amount of research is now being done to formulate better MAO inhibitors and design more rigorous clinical trials.^225^ It is likely, though, that these new efforts will continue to be plagued by the lack of MAO inhibitor specificity for the systems most strongly implicated in AD dysfunction, especially if NE misbalances between different regions of the brain prove clinically significant. This work does leave open, however, the idea that region‐specific NE stimulation, possibly through a combination of approaches to be discussed later in this review, may prove more effective.

Despite the current failure of MAO inhibitors, methylphenidate, a potent stimulant that acts as a norepinephrine‐dopamine reuptake inhibitor (NDRI) has been shown to slightly improve the attention and apathy of AD patients after symptom onset, demonstrating increased NE levels can result in a clinical benefit.^229^, ^230^ Studies are still extremely limited, and it has not been trialed as a preventative measure.^229^ A more targeted noradrenergic therapy is atomoxetine, a norepinephrine reuptake inhibitor (NRI), that acts as a relatively selective inhibitor of NET in the central nervous system, especially the forebrain.^231^ Multiple animal models have indicated possible therapeutic effects of atomoxetine, but clinical trials have not yet shown efficacy.^232^ It is possible that the late‐stage increase in forebrain NE is not enough to reverse or significantly alter the underlying pathology. In essence, previous approaches have proven to be too little, too late.

### 4.3 Targeted locus coeruleus therapies

There is a large body of research demonstrating an important and primary role of the noradrenergic system and LC remodeling in AD. Coupled with the lack of tangible results from existing noradrenergic therapies, it is important to ask whether the LC itself can be directly stimulated. Animal studies have shown that chemogenetic stimulation of the LC in transgenic TgF344‐AD rats resulted in the rescue of impaired reversal learning in a Morris water maze task.^233^ To‐date, no studies of direct LC stimulation have been performed in humans, but indirect modulation through non‐invasive brain stimulation (NIBS) techniques have been implemented in at least one clinical study (Figure 2). This pilot study using vagal nerve stimulation (VNS) was able to show that the treatment was well tolerated and that after one year 7 of 17 patients improved and 12 of 17 patients did not decline from baseline.^234^ Despite such initial promise, the mechanism of brain stimulation in AD is not well understood, and treatments remain primarily open loop with no adjustment possible for an individual's response to stimulation.^235^ Follow‐up clinical trials have not been widely pursued, and the mechanisms underlying the effect of VNS stimulation on AD, as well as the relationship with the LC, are not well understood. Work using VNS in animal models, however, has moved forward in exploring a variety of proposed mechanisms and will be discussed in the final section of this review (Supplementary Table 2C).

## 5 ADRENERGIC AND CHOLINERGIC RELATIONSHIP

An important connection that has been largely overlooked is the interaction between the noradrenergic and cholinergic centers of the brain. With the shift in AD focus from the NBM to the LC, there has been limited work understanding the impact of dysfunction of one of these systems on the other. One of the first studies to investigate this relationship, involved lesioning the fornix in rats, a structure important for transmitting acetylcholine to the hippocampus, and a subsequent neurochemical analysis of hippocampal tissue. It was found that choline acetyltransferase (ChAT) activity was reduced by 50%, correlating negatively with the number of errors the rats made in a maze task, while hippocampal NE also decreased by 50%.^236^ Interestingly, there was no decrease, and in fact a slight increase in the noradrenergic metabolite methylhydroxyphenylglycol (MHPG), suggesting a net increase in NE turnover despite the decrease in noradrenergic cells. These researchers postulated that the negative correlation between ChAT activity and NE turnover suggested that hyperactivity in the remaining noradrenergic neurons inhibited proper functioning of the remaining cholinergic neurons.^236^ This is a remarkably similar pathology to that seen in AD and warrants further exploration. It is important to note that the suspected mechanism of noradrenergic inhibition on the cholinergic system operated through the α_2_ receptors located on cholinergic terminals.^236^, ^237^, ^238^, ^239^

The dynamic relationship between these two neuromodulatory systems remains substantially uncharacterized, and limited work has been done explaining the precise mechanisms underlying adrenergic hyperactivation following LC disruption. There is evidence, however, that many of the mechanisms beneficial to memory storage occur with moderate levels of NE, while higher levels tend to impair memory storage.^240^ Additional work has shown that more substantial and specific lesions to the LC do indeed result in an increase in cortical release of ACh.^239^ This is likely not applicable to late‐stage AD when a significant portion of both the LC and NBM have degenerated and tau pathology has spread throughout the brain. It does raise interesting questions though about how this dysregulation can be targeted early in the course of AD.

Further compounding this relationship is additional evidence that decreased ACh activity possibly decreases noradrenergic tone.^241^ This implies that when AD reaches the point of significant cholinergic destruction, there would be a rapid deterioration in the previously compensated noradrenergic system. Again, the deleterious mechanisms of NE appear to be regulated through α_2_ ARs, while the memory enhancing effects appear to be mediate through the β ARs.^240^ This makes sense in the context of the relative affinities of each of these receptors. α_2_ ARs have the highest binding affinity to NE, while β ARs have the lowest.^173^ Taken together with the information previously presented on the overall noradrenergic dysfunction seen in AD, this likely accounts for some of the contradictory effects of NE seen in experiments. Successful treatment approaches are likely to be ones that can stimulate healthy LC function before significant dendritic remodeling occurs, with an emphasis on immune regulation and perhaps selective α_2_ blockade.

## 6 THE FUTURE OF ELECTROCEUTICAL THERAPIES IN ALZHEIMER'S DISEASE

Direct neural stimulation offers many interesting and unique opportunities to halt or reverse AD pathology.^56^, ^57^, ^58^, ^59^, ^60^, ^61^, ^62^, ^63^, ^64^, ^65^, ^66^, ^67^, ^68^, ^69^, ^70^, ^71^, ^72^, ^73^ In addition to the need to further develop the NBM‐DBS approach mentioned above, there are also a variety of theoretically viable methods that can capitalize on a new AD therapeutic approach centered on the LC or entorhinal cortex (Figure 2).^242^, ^243^ Vagus nerve stimulation promises several unique, non‐invasive approaches to many of the hypotheses for AD, and can be applied at nearly every stage of the disease. The last clinically significant trial with this technology took place nearly two decades ago, and the control techniques available to researchers and physicians have improved substantially since that time. A tremendous amount of work remains to explore the underlying mechanisms and further develop the technology needed to address each of these promising avenues.

**FIGURE 4:**
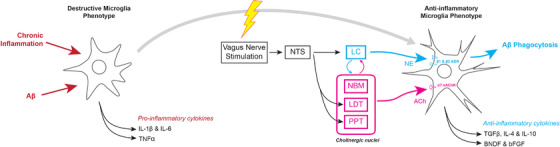
Anti‐inflammatory mechanisms of vagus nerve stimulation. Stimulation of the vagus nerve can result in the activation of anti‐inflammatory signaling cascades that shift microglia towards a phenotype more adept at clearing AD pathology.

Vagal nerve stimulation (VNS) (Supplementary Table 2C) has been used for many years to treat various neurological and psychological conditions, including major depressive disorder and epilepsy. It is a safe and effective technique that allows a minimally invasive interface with the adrenergic system and, to a lesser known extent, the cholinergic system.^244^, ^245^, ^246^ Experimental models of LC degeneration have shown that significantly decreased levels of NE can suppress microglial phagocytosis of beta amyloid (Figure 4).^191^, ^192^, ^193^, ^194^ VNS offers an opportunity to restore substantial microglial function in late stage AD, where NE levels may be substantially decreased in specific brain regions.

Combined with early detection markers currently under development, VNS stands out as a relatively early intervention, when accumulating neurotoxic products elicit a generalized inflammatory response that further exacerbates neurodegeneration and dysfunction of the cerebrovascular endothelium.^247^, ^248^ VNS stimulation, in contrast to pure NE manipulation, has been shown to significantly reduce plasma levels of tumor necrosis factor‐α and prevent hippocampal microglial activation (Figure 4).^249^, ^250^ Much of this additional benefit from VNS may arise from simultaneous activation of what is known as the cholinergic anti‐inflammatory pathway, which is implicated in encouraging a neuroprotective microglial phenotype through activation of the α_7_ subtype of the nicotinic acetylcholine receptor.^250^, ^251^ Recent research has shown that microglial activation appears to be both a specific response to early Aβ plaque deposition as well as a nonspecific and late response to subsequent neurodegeneration.^252^ VNS may be able to modulate the immune system in both early and late stages of the disease. This could perhaps delay symptomatic manifestations of AD to a point where they are no longer clinically relevant.

Given the dendritic remodeling discussed previously in this review, there is a need to better characterize the functional ability of VNS to either prevent or selectively reverse this change in AD patients. Previous work has shown that VNS is capable of modulating the dendritic cell profile outside of the central nervous system.^253^, ^254^ In the central nervous system, it is also known that VNS can induce neuronal plasticity.^255^, ^256^ Eventual control over these processes offers significant therapeutic potential, but the work is too pre‐mature to comment on in‐depth during this review.

## 7 CONCLUSION

The role NE plays in AD remains complicated. Both VNS and experiments with direct LC stimulation present opportunities to assess if the neuroprotective factors of NE remain true through all stages of AD. VNS is well documented to increase the extra‐cellular levels of NE in the hippocampus and cortex, depending on the intensity of stimulation.^257^ This response can be used to evaluate an intervention that increases endogenous NE levels in a method that mimics natural LC communication.

VNS as an intervention in both early and late AD should be investigated more in‐depth. The invasive methods of directly stimulating the LC can also help differentiate the role of NE specifically, as opposed to the more general effect of VNS in the forebrain, thalamus, and reticular formation.^258^ This non‐specific, generalized response to VNS may hold promise for reducing the cognitive deficits in AD patients through an entirely unrecognized set of mechanisms. It is imperative that researchers continue constructing methods to more accurately control the stimulation and response of both peripheral and central neural stimulation. A more nuanced understanding of the time‐dependent changes across AD in relation to the LC, with and without VNS, offers an opportunity to substantially increase our understanding of this devastating disease and to develop more effective therapeutics.

## Supporting information

Table S1

Table S2
