# Supplementary material for: Alzheimer's disease: An evolving understanding of noradrenergic involvement and the promising future of electroceutical therapies
Source: Clin Transl Med. 2021 May 1;11(4):e397. doi: 10.1002/ctm2.397 (PMC8087948; doi:10.1002/ctm2.397)
Supplement: Supplementary file 1 — Table S1 [file CTM2-11-e397-s002.docx]

# Supplementary Table 1. Timeline and Clinical Impact of Current Alzheimer’s Disease Hypotheses

| Year | Author | Hypothesis | Clinical Impact |
| --- | --- | --- | --- |
| 1976 | P Davies, AJ Maloney | Cholinergic Hypothesis | Cholinesterase inhibitors have been shown to have a consistent, though marginal, increase in functional outcomes over placebo.^48, 54^ Pilot studies using invasive NBM stimulation have shown evidence of disease stabilization.^56-59, 74, 75^ |
| 1991 | J Hardy, D Allsop | Amyloid cascade hypothesis | No Aβ-clearing therapies have yet been shown to confer major clinical benefit.^97^ Aducanumab, a monoclonal IgG1 antibody, has shown minor clinical benefit, with phase 3 trials ongoing until 2022.^45, 97, 276-278^ |
| 1994 | L Buée et al | Neurovascular Hypothesis | Multiple clinical trials using statins, diabetic medications, and anti-hypertensives have failed to show benefit in AD.^115-117^ Though contributary, it is increasingly unlikely neurovascular dysfunction is the primary driver of AD.^45^ |
| 2004 | RH Swerdlow, SM Khan | Mitochondrial cascade hypothesis | Clinical trials have focused on antioxidants such as idebenone and triphenylphosphonium, with only the latter showing consistent results in animal models.^128-131^ Future work appears to focus on nanoparticle delivery directly to mitochondria, with clinical benefit unknown.^132-135^ |
| 2009 | B Frost, RL Jacks, MI Diamond | Tau propagation hypothesis | Early efforts at microtubule stabilization, inhibition of the kinases responsible for phosphorylating tau, or direct inhibition of tau aggregation have not yet led to clinically useful therapies.^39^ Numerous tau immunotherapies have shown promising safety profiles and clinical trials are scheduled to continue throughout the early 2020s.^39, 45, 153, 154^ |
