# Supplementary material for: Alzheimer's disease: An evolving understanding of noradrenergic involvement and the promising future of electroceutical therapies
Source: Clin Transl Med. 2021 May 1;11(4):e397. doi: 10.1002/ctm2.397 (PMC8087948; doi:10.1002/ctm2.397)
Supplement: Supplementary file 2 — Table S2 [file CTM2-11-e397-s001.docx]

# Supplementary Table 2. Summary of Relevant Literature

| A. Literature Concerning the Relationship Between Norepinephrine and Alzheimer's Disease | | | |
| --- | --- | --- | --- |
| Year | **Author** | **Title** | **Key Findings** |
| 1981 | BE Tomlinson et al | Cell loss in the locus coeruleus in senile dementia of Alzheimer type | Among cognitively intact individuals, there is a gradual reduction of pigmented cells in the LC from middle to old age. The mean count of cells in old patients with senile dementia of Alzheimer type was significantly less than the cognitively intact older group.^26^ |
| 1983 | L Iversen et al | Loss of pigmented dopamine-beta-hydroxylase positive cells from locus coeruleus in senile dementia of Alzheimer's type | Patients with senile dementia Alzheimer type exhibited an average loss of about 60% in locus coeruleus cells compared to age-matched controls. There was no significant change LC cell count in patients with a different form of dementia.^156^ |
| 1993 | SM Tejani-Butt et al | Norepinephrine transporter sites are decreased in the locus coeruleus in Alzheimer's disease | A significant decrease in [3H]nisoxetine binding to NE transport sites is seen in the mid and caudal regions of the LC in AD, possibly due to loss of NE transporter sites on the terminals of noradrenergic neurons in the LC.^279^ |
| 2003 | C Zarow et al | Neuronal loss is greater in the locus coeruleus than nucleus basalis and substantia nigra in Alzheimer and Parkinson diseases | In AD, neuronal loss was most severe in the LC and NBM. The duration of illness was better correlated with LC degeneration than NBM degeneration.^157^ |
| 2006 | M Haglund et al | Locus ceruleus degeneration is ubiquitous in Alzheimer's disease: possible implications for diagnosis and treatment | LC degeneration is significantly more severe in AD than in vascular dementia. The authors suggest further study into the consequences of noradrenergic dysfunction in AD.^280^ |
| 2006 | MT Heneka et al | Locus ceruleus degeneration promotes Alzheimer pathogenesis in amyloid precursor protein 23 transgenic mice | Chemically induced LC degeneration in APP23 transgenic mice resulted in significantly increased neurodegeneration and neuronal loss. Brain regions without LC projections remained unaffected. LC degeneration is postulated to substantially contribute to AD development.^155^ |
| 2007 | A Grudzien et al | Locus coeruleus neurofibrillary degeneration in aging, mild cognitive impairment and early Alzheimer's disease | Tissue samples from seven cognitively normal individuals and five subjects with MCI or early AD showed significantly higher levels of abnormal tau in the LC of cognitively impaired individuals. There was a negative correlation between Mini Mental Status Exam scores and markers of tau pathology in the LC.^27^ |
| 2010 | B Gulyas et al | The norepinephrine transporter (NET) radioligand (S,S)-[18F]FMeNER-D2 shows significant decreases in NET density in the human brain in Alzheimer's disease: a post-mortem autoradiographic study | Patients with AD demonstrate a significant decrease in norepinephrine transporter density in both the LC and thalamus compared to age-matched controls. These decreases correlate with the Braak grade assigned to each patient’s disease progression.^189^ |
| 2010 | MT Heneka et al | Locus ceruleus controls Alzheimer's disease pathology by modulating microglial functions through norepinephrine | Induced degeneration of the LC in APP-transgenic mice increases expression of inflammatory mediators, resulting in elevated Abeta deposition and reduced recruitment of microglia to Abeta plaque sites. Supplementation of NE precursor L-threo-DOPS restores microglial function. NE stimulation of mice microglia suppress Abeta-induced cytokine/chemokine production and increase microglial migration and phagocytosis of Abeta.^194^ |
| 2017 | SC Kelly et al | Locus coeruleus cellular and molecular pathology during the progression of Alzheimer's disease | Post-portem tissue samples were collected from subjects with no cognitive impairment (NCI), amnestic cognitive impairment (aMCI), and mild/moderate AD. Estimates of total LC number showed a 30% loss during the transition from NCI to aMCI, and an additional 25% loss from aMCI to AD. Reduction in LC neuron number was also associated with increased postmortem neuropathology.^160^ |
| 2017 | JM Rorabaugh et al | Chemogenetic locus coeruleus activation restores reversal learning in a rat model of Alzheimer's disease | In an APPsw transgenic rat model (TgF344-AD), hyperphosphorylated tau is detected in the LC prior to accumulation in the medial entorhinal cortex or hippocampus. Tau pathology in the LC of these animals is negatively correlated with noradrenergic innervation in the medial entorhinal cortex. They also demonstrate progressive loss of hippocampal NE levels and LC cells in the medial entorhinal cortex and dentate gyrus. Impaired reversal learning was rescued by chemogenetic activation of the LC.^233^ |
| 2017 | P Theofilas et al | Locus coeruleus volume and cell population changes during Alzheimer's disease progression: A stereological study in human postmortem brains with potential implication for early-stage biomarker discovery | Unbiased sterological analyses of human brainstems revealed an 8.4% reduction of LC volume for every unit increase in the Braak stage. Neuronal loss started midway through AD progression, and age-related changes spare the LC.^24^ |
| 2019 | M Gannon, Q Wang | Complex noradrenergic dysfunction in Alzheimer's disease: Low norepinephrine input is not always to blame | This review postulates that the loss of integrity of the noradrenergic system, due to adaptive changes of the LC in AD, is a key driver pathogenesis. Simple loss of NE input appears unable to fully explain disease progression given mixed data supporting sustained extracellular NE levels in the cortex late in AD.^170^ |
| 2019 | HIL Jacobs et al | Alzheimer's disease pathology: pathways between central norepinephrine activity, memory, and neuropsychiatric symptoms | Neuropsychiatric symptoms in AD patients showed a strong correlation to both MHPG (NE metabolite) and p-tau. Memory deficits are linked to MHPG via a combination of p-tau and amyloidosis, suggesting the LC-NE system is central in the link between AD pathology and its related behavioral/cognitive deficits.^220^ |
| B. Literature Concerning Vagal Nerve Stimulation for Neurodegenerative Diseases | | | |
| Year | **Author** | **Title** | **Key Findings** |
| 2006 | CA Merrill et al | Vagus nerve stimulation in patients with Alzheimer's disease: Additional follow-up results of a pilot study through 1 year | VNS was administered to 17 AD patients for 1 year and resulted in 12 patients either improving or not declining, as measured by the Alzheimer’s Disease Assessment Scale-cognitive subscale and Mini Mental Status Exam. The median change in CSF tau at 1 year was a reduction of 4.8%. VNS for AD is safe and well-tolerated for long-term administration.^234^ |
| 2006 | RW Roosevelt et al | Increased extracellular concentrations of norepinephrine in cortex and hippocampus following vagus nerve stimulation in the rat | 1.0 mA stimulation of the left vagus nerve at the cervical level resulted in increased NE concentrations in the cortex and hippocampus bilaterally, while 0.5 mA stimulation only increased NE in the hippocampus. The NE increases were transient and confined to the stimulation periods.^257^ |
| 2009 | F Biggio et al | Chronic vagus nerve stimulation induces neuronal plasticity in the rat hippocampus | Chronic VNS induces a long-term increase in brain-derived neurotrophic factor (BDNF) immunoreactivity and the number of BDNF+ cells. The dendritic complexity of doublecortin neurons in the hippocampus also increases. Behavior changes in this experiment were not evident.^255^ |
| 2016 | DR Hulsey et al | Reorganization of Motor Cortex by Vagus Nerve Stimulation Requires Cholinergic Innervation | VNS paired with forelimb training in rats results in an increase in the representation of that forelimb when the NBM is intact. NBM lesion prevents this VNS-dependent increase in representation.^245^ |
| 2017 | R Kaczmarczyk et al | Microglia modulation through external vagus nerve stimulation in a murine model of Alzheimer's disease | Non-invasive VNS administration in APP/PS1 transgenic mice resulted in the morphologic change of microglia from a neurodestructive phenotype to a neuroprotective phenotype.^250^ |
| 2019 | WJ Huffman et al | Modulation of neuroinflammation and memory dysfunction using percutaneous vagus nerve stimulation in mice | Minimally invasive, targeted VNS in mice reduced plasma levels of tumor necrosis factor-alpha 3 hours post injection of lipopolysaccharide (LPS). VNS also prevented LPS-induced microglial activation in the hippocampus.^249^ |
| 2020 | C Liu et al | Neural networks and the anti-inflammatory effect of transcutaneous auricular vagus nerve stimulation in depression | Transcutaneous auricular VNS can significantly modulate the activity and connectivity of a wide range of neural networks involved in emotion, reward, and executive function. A link between suppression of inflammation post VNS and changes in brain circuitry is proposed to exist in this review.^247^ |
| C. Literature Concerning Deep Brain Stimulation for Alzheimer's Disease | | | |
| Year | **Author** | **Title** | **Key Findings** |
| 2015 | J Kuhn et al | Deep brain stimulation of the nucleus basalis of Meynert in Alzheimer's dementia | Four of six patients were considered responders at 11-month follow-up, based on stable or improved Alzheimer’s Disease Assessment Scale-cognitive subscale scores. No adverse outcomes were noted.^56^ |
| 2016 | K Hardenacke et al | Deep Brain Stimulation of the Nucleus Basalis of Meynert in Alzheimer's Dementia: Potential Predictors of Cognitive Change and Results of a Long-Term Follow-Up in Eight Patients | Long-term follow-up of 8 NBM-DBS patients at 24 months suggests intervention at earlier an earlier stage of the disease has a favorable impact on overall disease progresssion.^57^ |
| 2016 | AM Lozano et al | A Phase II Study of Fornix Deep Brain Stimulation in Mild Alzheimer's Disease | Bilateral DBS in 42 patients with mild AD was overall well-tolerated and associated with an increase in cerebral glucose metabolism. Patients over 65 years saw a possible benefit, while those less than 65 may have experienced a possible decline.^60^ |
| 2018 | M Aldehri et al | Deep brain stimulation for Alzheimer's Disease: An update | A review of studies from December 2013 to March 2017 revealed that DBS for AD appears to be most beneficial for patients who are in the early stages of the disease. Potential mechanisms include hippocampal enlargement and enhanced neurotransmitter release. Conclusions about the applicability of DBS in AD are still premature.^62^ |
| 2018 | J Baldermann et al | Neuroanatomical Characteristics Associated With Response to Deep Brain Stimulation of the Nucleus Basalis of Meynert for Alzheimer's Disease | MRI scans of ten AD patients that received NBM-DBS indicate that patients with less severe atrophy may see benefits from the treatment. These beneficial effects are related to the preservation of fronto-parieto-temporal connections.^63^ |
| 2018 | J Leoutsakos et al | Deep Brain Stimulation Targeting the Fornix for Mild Alzheimer Dementia (the ADvance Trial): A Two Year Follow-up Including Results of Delayed Activation | DBS stimulation in AD patients is safe over two years, but additional exploration of the underlying mechanisms and treatment strategies should be further explored to determine if DBS can be used as a treatment in AD.^65^ |
| 2018 | A Mann et al | Chronic deep brain stimulation in an Alzheimer's disease mouse model enhances memory and reduces pathological hallmarks | DBS of the entorhinal cortex in AD mice normalized their impaired performance in a Morris water maze task, with an associated increase in neurogenesis in the dentate gyrus, reduction in beta-amyloid-plaques, and decreased cortical tau.^67^ |
| 2018 | Z Mao et al | Partial improvement in performance of patients with severe Alzheimer's disease at an early stage of fornix deep brain stimulation | Fornix-DBS in five patients with AD resulted in improvement of some cognitive functions, as well as emotional and social performance, in an early stage of the disease. Long-term effects need to be further validated.^68^ |
| 2018 | D Scharre et al | Deep Brain Stimulation of Frontal Lobe Networks to Treat Alzheimer's Disease | DBS at the ventral capsule/ventral striatum in three patients with AD was well-tolerated and resulted in less performance decline as measured on the Clinical Dementia Rating-Sum of Boxes. Further work should be done to utilize frontal network modulation to improve executive function in AD.^70^ |
